# Altered visual feedback from an embodied avatar unconsciously influences movement amplitude and muscle activity

**DOI:** 10.1038/s41598-019-56034-5

**Published:** 2019-12-24

**Authors:** Pierre Bourdin, Matteo Martini, Maria V. Sanchez-Vives

**Affiliations:** 1 Institut d’Investigacions Biomèdiques August Pi i Sunyer (IDIBAPS), Barcelona, Spain; 2 Experimental Virtual Environments for Neuroscience and Technology (EVENT) Laboratory, Department of Clinical Psychology and Psychobiology, University of Barcelona, Barcelona, Spain; 3 Institució Catalana Recerca i Estudis Avançats (ICREA), Barcelona, Spain; 4 EIMT, Universitat Oberta de Catalunya, Barcelona, Spain; 5 Department of Psychology, University of East London, London, UK

**Keywords:** Cognitive neuroscience, Psychology

## Abstract

Evidence suggests that the sense of the position of our body parts can be surreptitiously deceived, for instance through illusory visual inputs. However, whether altered visual feedback during limb movement can induce substantial unconscious motor and muscular adjustments is not known. To address this question, we covertly manipulated virtual body movements in immersive virtual reality. Participants were instructed to flex their elbow to 90° while tensing an elastic band, as their virtual arm reproduced the same, a reduced (75°), or an amplified (105°) movement. We recorded muscle activity using electromyography, and assessed body ownership, agency and proprioception of the arm. Our results not only show that participants compensated for the avatar’s manipulated arm movement while being completely unaware of it, but also that it is possible to induce unconscious motor adaptations requiring significant changes in muscular activity. Altered visual feedback through body ownership illusions can influence motor performance in a process that bypasses awareness.

## Introduction

Human perception can be easily challenged by afferent cross-modal stimulation in a way that leads to illusory beliefs. One striking example, the “rubber hand illusion” has triggered an increasing number of investigations over the last twenty years^1^. According to this illusion, perceiving a tactile stimulation on one’s own hidden hand while seeing a prosthetic hand stroked synchronously, produces in the majority of participants, the illusory sensation that the prosthetic limb belongs to one’s own body. Such multisensory stimulation has been widely exploited to reproduce a feeling of ownership over both prosthetic and virtual limbs (see, e.g.,^2–4^). In addition, synchronous visuotactile stimulations in rubber hand illusion paradigms have been shown to yield another perceptual illusion known as “proprioceptive drift”^5^. This illusion entails a remapping of the perceived own hand position, which results to be felt closer to the rubber hand^1^. Illusory perceptions such as the “proprioceptive drift” are examples of how the internal representation of our body in space is highly plastic and depends on congruent multisensory afferent signals to maintain itself as a stable and coherent unit. But how can healthy individuals perceive a part of their body located at a certain place when it is not actually there? A simple explanation to this question derives from the notion that when different sensory modalities convey conflicting information, like in the classical rubber hand paradigm, visual information about hand location can dominate proprioception^6^, leading to a mislocalization of one’s own limb. An example of this misleading process can be found in an experiment concerning the recalibration of the limb position conducted by Newport and Gilpin^7^. In this study, the authors asked participants to hold their hands in a stationary position at a constant distance from each other inside the MIRAGE box, a multisensory illusion device which presents live video images of their real, hidden, hands. Visual feedback manipulation of the hand images led participants to believe that their hands were getting closer to each other although they were not. In response to this altered visual feedback, to compensate for the seen position, participants slightly moved their hands away from each other. Thus, when participants were asked to reach their right-hand with the left one, after the right-hand image disappeared from the view, they reached an empty space, therefore getting the illusion that their right hand had disappeared. Using an identical set-up and a similar procedure, Bellan and coworkers investigated how vision and proprioception interact over time with the localization of one’s own hidden hand^8^. In this experiment participants were asked to report their right-hand position by verbally stopping a cursor that was moving on top of the screen, when it reached the position where they thought their hand was. The authors showed that, to complete the task, participants initially relied more on visual memory, disregarding the information coming from their proprioception and thus failing to correctly localize their hand. However, over time, the reliance on proprioceptive information increased, hence improving their hand localization. In addition, the authors found that the switch to proprioception occurred earlier when participants were asked to close their eyes; that is, when the visual memory of the hand positions faded^8^. In a similar recent study, Abdulkarim and Ehrsson investigated active versus passive imperceptible movements using a setup displaying a live video feed of the hand^9^, which led to an unconscious adjustment of the participants’ right-hand position. In their active condition, the sensorimotor illusion caused by the altered visual feedback prompted the participants to actively (but unknowingly) displace laterally their unseen right hand, whereas in the passive condition, participants’ hand was mechanically displaced at a slow, unnoticeable velocity. The authors found that during active displacement, the location of the hand indicated by the participants was closer to the position indicated on the screen, while in the passive condition, the location indicated by participants was closer to the real hand location^9^. These studies suggest that vision predominates over proprioception in limb localization tasks, and that the reliance on altered visual input can increase the error in limb position estimation. Importantly, such empirical evidence also reveals that fine-tuned visuomotor adaptations can be unconscious. In addition, although information generated by muscle spindles contributes significantly to the sense of position and movement^10^, it remains unclear whether the level of muscle activity is important for the awareness of the precise position of our own limb. In 2008, Slater *et al*. showed that the vision of a virtual arm falling off a virtual table was enough to induce electromyographical activity in the participant's real arm, and that the level of this activity correlated with the subjective feeling of ownership over the virtual arm^4^. Nonetheless, to our knowledge, no studies have so far investigated the contribution of muscular activity to limb position awareness. The role of limb position awareness during movement is not only relevant for psychophysiology but it also has clinical implications for rehabilitation. Indeed, rehabilitation practices often involve movements of the affected limb under a range of muscular contraction levels^11,12^, and it is known that providing patients with covert illusory feedback may improve rehabilitation outcomes^13,14^. Therefore, in the current study we investigated whether altered visual feedback during movement of an embodied avatar’s arm could induce significant range of motion and muscular activation adjustments of the real arm, as indicated by the active range of elbow flexion and biceps electromyography, respectively. Crucially, we also wanted to assess whether these muscular adjustments can elude awareness. For this we used immersive virtual reality (VR), a powerful tool to induce illusory perceptions. In VR both the environment and the human characters (i.e. avatars) can be modified according to the experimenter’s need, while still benefitting of a sound methodological approach^15,16^. Here we used a virtual body ownership paradigm^17^ to provide an altered visual feedback of the limb position, by introducing a variable drift between the virtual and the real arm. In three conditions, we modified the drift of the virtual arm with respect to the real one so that when the real arm was flexed to 90° at the elbow (against resistance using a rubber band), the virtual arm appeared flexed to either a smaller, the same or a larger angle. We measured participants’ motor performance by recording both the actual position of the real arm using a motion tracking system and electromyographic (EMG) activity of the right biceps muscle. Surface EMG recordings allowed us to evaluate muscle recruitment, having previously been shown to be efficient for monitoring muscle activity during dynamic tasks^18^.

We hypothesized that a surreptitious reduced or amplified movement of the virtual limb would lead participants to respectively increase or decrease the real arm movement’s amplitude, consequently altering the level of muscular activation. Our final aim was to see whether significant changes in motor behavior of participants could go unnoticed. 

## Results

### Arm range of motion

**Figure 1:**
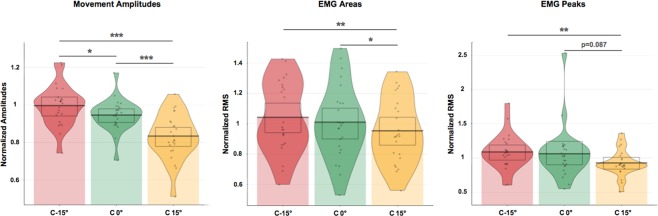
“Pirate plots” representing movement amplitudes, normalized root mean squares (RMS) of EMG areas and EMG peaks, per each condition (“C-15°”, “C 0°”, “C 15°”). Single points depicts raw data, the bar lines the means, the so-called ‘beans’ (or smoothed density curves) show the data full distribution, and the ‘bands’ (boxes) the confidence intervals. Asterisks denote significant differences between conditions (*p < 0.05; **p < 0.01; ***p < 0.001).

The altered visual feedback provided by the virtual arm had a significant effect on the range of real arm’s movement (*F*_*(*2*,46)*_ = 40.48; *p* < 0.001, *η*^2^ = 0.64). Specifically, the condition where the avatar’s arm was reduced by an artificial drift (“C −15°”), led to a significantly larger real arm movement as compared to both the condition where no drift was applied (“C 0°”; *p* = 0.026), and the condition where the course of the avatar’s arm was increased by a drift (“C 15°”; *p* < 0.001). Furthermore, the condition where the avatar’s arm movement was amplified (“C 15°”), led to a significantly smaller movement of the real arm in comparison to the no-drift condition (“C 15°” vs. “C 0°”: *p* < 0.001, see Fig. 1).

### Muscular activity (EMG)

As we observed for the arm movement amplitudes, the altered visual feedback led to significantly different magnitudes of the real arm’s muscular activity, as shown by the index of the EMG peaks (*F*_*(*2*,46)*_ = 4.80; G-G corrected *p* = 0.022; *η*^2^ = 0.17), especially between the conditions “C 15°” and the “C −15°”, with the latter inducing stronger muscular activations (EMG peaks: “C 15°” vs. “C −15°”: *p* = 0.001). Also, in the condition in which the avatar’s arm range of movement was increased (“15°”) the EMG peaks were on average smaller than the ones reported in the condition where the avatar’s arm had no drift (“C 15°” vs. “C 0°”: *p* = 0.087). These results were confirmed by the index of the overall muscular activity (i.e. EMG areas) for which a large effect of condition was found (*F*_*(*2*,46)*_ = 9.70; *p* < 0.001, *η*^*2*^ = 0.30); post-hoc tests revealed significantly stronger muscular activity in the “C 0°” and “C-15°” conditions compared to the “C 15°” one (respectively *p* = 0.029 and *p* = 0.001). Not only did participants subconsciously perform a larger arm movement in the reduced drift condition, they also exerted more force, since the movement was executed against a more-tense elastic band. Therefore, under covert visual feedback manipulations, participants on average regulated their muscle activity differently to accomplish the apparently unchanged motor task. No other comparisons were significant (all *p* > 0.05).

### Subjective ratings

**Figure 2:**
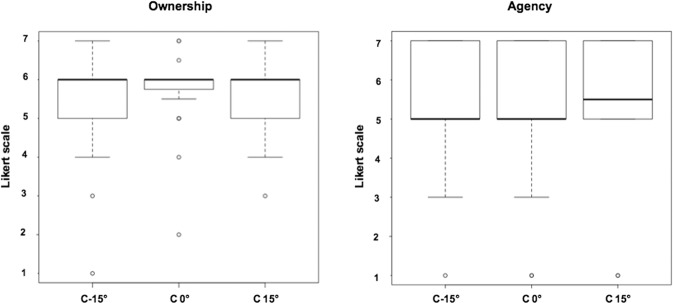
Box plots of ownership and agency ratings during each condition. Both items were scored according to a 7-point Likert Scale, 1 representing “Not at all” and 7 “Totally”.

The levels of both agency and ownership over the virtual arm were high and did not change significantly among conditions (see Fig. 2). Indeed, the Friedman ANOVAs did not show any significant effect of condition on ownership (*χ*^*2*^ = 0.61; *p* = 0.73) or agency (*χ*^*2*^ = 2.00; *p* = 0.37). The avatar’s arm was consistently strongly felt as belonging to their real body and fully controlled as if it was their own real limb.

### Awareness check

At the end of the experiment, participants were asked whether they noticed any experimental manipulation between the different conditions, and in particular whether they had noticed any change in the avatar’s arm position and movement compared to their real arm. Importantly, they were also asked whether they noticed any change in the way they were performing the task. Three participants out of 27 (i.e. representing the 11%) were excluded from the study because they noticed that the avatars’ movements could have been altered. All included participants reported that they always performed the same movements throughout the experiment (i.e., their perceived movement amplitude and force exerted was always the same) and did not notice any experimental manipulations or differences between conditions.

## Discussion

In the present study we assessed whether altered visual feedback while moving an embodied virtual avatar’s arm could induce unaware motor adjustments of the real arm. Not only did we find that the sense of limb position is heavily influenced by visual feedback during movement, but also that force adjustments can be completely unconscious. Furthermore, our results show that such unconscious motor adjustment can occur in the majority of the population. Indeed, from our initial sample, only 3 participants out of 27 suspected that the avatars’ movements could have been altered, while all the other participants did not notice any manipulations. The compensatory motor activity observed in our experiment backs the idea of a predominance of vision over proprioception during motor tasks with altered visual feedback^19,20^. We demonstrate that this visual dominance sets in unconsciously even during large voluntary movements of limbs, where motor adjustments require significantly different levels of muscular activity.

Unconscious motor adjustments have already been documented in previous studies. For instance, in a study conducted by Varraine and colleagues^21^ the authors showed how participants were unaware of force increase while walking on a treadmill at different levels of resistance. However, the limited sample size (*n* = 6), the absence of a visual manipulation and the lack of measurement of the muscular activity limit the insights that can be derived from their experiment.

Our findings suggest that individuals can reach, without noticing, different levels of muscle recruitment during voluntary limb movements, and confirm that the incongruence between the proprioceptive and the visual feedback can be largely overlooked in favour of the visual input. These types of visual manipulations, which can surreptitiously drive motor behaviour, may turn to be useful in rehabilitation settings, where voluntary movements, visual feedback and motivation play an important role in many treatment protocols^22^. After all, illusory visual feedback has already been shown to represent a useful tool in the clinical practice; it is effective in reducing pain perception during chronic^23–25^ or experimentally induced pain states^26–29^, and it has been proven to be useful for motor rehabilitation in stroke patients^30–32^.

Poor motor awareness has been most often associated to individuals with neurological disorders, but musculoskeletal injuries also result in motor and proprioceptive deficits through a combination of afferent disruption, limb disuse and altered cortical representations. For instance, previous studies with patients reported an impaired motor awareness whenever a conflict between the actual limb movement and the visual feedback occurred^33,34^. Also, motor awareness has been shown to be affected in patients with anosognosia for hemiplegia and motor neglect^35^. However, poor motor awareness seems to affect not only people with neurological and musculoskeletal problems, but it has been documented in healthy individuals too. For instance, in a study conducted by Fourneret & Jeannerod it was found that when tracing sagittal lines with their right hand, normal subjects compensated for the visual distortion introduced in some trials, but they systematically underestimated the hand deviation. The poor conscious monitoring of motor performance was confirmed when the same subjects were asked to indicate the perceived direction of their hand by drawing a line with their eyes closed^36^. Along these lines, the current study provides direct evidence that, at least under specific experimental conditions, participants can significantly modulate their movement amplitude and muscle activity to accomplish a task, being completely unaware of such accommodation. Interestingly, motor awareness and perceptual awareness can be dissociated in a double-step task^37^. Classically this task concerns rapid pointing movements towards a visual target, without vision of the hand. While performing the task, the target is slightly shifted forcing the participants to correct their ongoing trajectories according to the direction of the visual target shift, but the participants remain unaware of such a change. This shows the brain systems generating motor awareness have access to information about the target location even if this information does not enter the perceptual awareness. Indeed, evidence deriving from neural activity highlights the importance of the integration between proprioceptive and visual inputs for determining the position of the hand during motor control^38^.

It has been suggested that motor awareness does not emerge from the sensory signals generated by the movement, but from the predictions of those sensory signals made in a feed-forward model^39,40^. In this experiment the prediction of the entire movement was kept constant in all conditions: the end position of the real arm was subjectively experienced at 90° with respect to the fixed starting position. Our findings show that the great majority of participants reported being unaware of any changes in their performed movement. Thus, these results are in agreement with the view that awareness of a movement is not influenced by the sensory output generated by the movement but, rather, by its prediction; and this despite the significant changes in their muscular activity shown by the electromyographic signal. Yet, when reliance on sensory output takes place, vision seems to lead motor control, until proprioception takes over when visual input is no longer available^8^.

Furthermore, in spite of the differences in movement amplitude and muscle activity, all VR conditions induced similarly high ratings of ownership towards the virtual limb. This is in agreement with previous findings that showed how the ownership of a virtual limb can be effectively induced by visuomotor correlations^41^. Further, it is coherent with the previous finding that larger levels of ownership of the virtual arm are associated with larger electromyographical activity associated with movements of the virtual arm^4^. Moreover, our participants did not detect any anomaly related to the avatar’s arm, which was continually felt as strongly belonging to their real body and fully controlled as if it were their own real limb. The visuomotor correspondence between the real and the virtual arm was also accompanied by a high level of agency, which implies that participants did not consciously experience any differences of control over the avatar’s arm across conditions. A check of the agency levels in our experiment was deemed to be important since it has been shown that the sense of agency can be strongly related to action awareness^42^. Therefore, in the present experiment, differences in the execution of the movement cannot be attributed to changes in the sense of agency or ownership. It is also important to notice however, that the relationship between action awareness and the sense of agency has been studied in the past within the context of the ‘temporal binding’ paradigm (for ex.^43–45^) which significantly differs from our task. Therefore, any conceptual parallelism with those studies should be made keeping in mind this caveat.

Finally, in clinical settings, VR provides novel and valuable resources for medical rehabilitation purposes, offering novel strategies for sensorimotor training in neurorehabilitation and musculoskeletal rehabilitation^46,47^. Visual feedback has been shown to be particularly powerful when it is delivered through VR, a technology that has shown to provide better patient training compared to traditional non-VR interventions^48^. Patients spend increasingly more time actively engaged in VR training^49^, and although VR-based therapies for motor function rehabilitation in post-stroke patients have produced some mixed results^50,51^ several studies showed its efficacy^52–58^. VR-based therapy efficacy could be boosted providing patients with covert illusory visual feedback, fostering the patient’s positive expectations, self-efficacy and suggestion^13,14^. Because body ownership illusions in immersive VR challenge the body-self relations^59–62^, the use of the “virtual body ownership” illusion may lead to further improvement in rehabilitation outcomes.

To summarize, the present study shows that it is possible to induce in healthy participants unconscious motor adaptations that require significant changes in muscular activity and result in reduced or amplified arm movements under a virtual body ownership paradigm. Although further studies are needed to explore the physical limits of these unconscious motor adaptations during altered visual feedback, our findings are relevant for the development and improvement of VR tools for clinical use.

## Methods

### Participants

Twenty-seven right-handed healthy participants (18 female, mean age ± SD 24 ± 9.34) with normal or corrected-to-normal vision and no history of neurological, motor or psychological disorders participated in this study. Their right-handedness was assessed with the Edinburgh handedness questionnaire^63^ before participating. Three out of 27 participants (11.11%: 2 males and 1 female) were discarded from all further analysis because, at the end of the experiment, they suspected that the arm movement was manipulated. Therefore, 24 participants (17 females) were finally included in the experiment (mean age 24 ± SD 9.33). The final sample size (*n* = 24) was estimated using G*Power and considering a medium effect size (*f*) of 0.25, an α error probability of 0.05, and a power (1- β error probability) of 0.8. All participants were fully informed about the experimental procedure apart from the manipulation operated on the avatar’s arm movements. Participants gave written informed consent, filled out a demographic information questionnaire before the start of the experiment and were paid 5 euros for their participation. The experimental protocol was approved by the local ethics committee (Comité Ético de Investigación Clínica de la Corporación Sanitaria Hospital Clínic de Barcelona) and the experiment was carried out in accordance with the Declaration of Helsinki.

### Apparatus

The Virtual Environment (VE) was designed and rendered with the Unity (http://unity3d.com) game engine. The avatars used for representing the virtual body of the participants were 3D models of male and female characters purchased from Rocketbox (http://www.rocketbox-libraries.com/) Studios. The VE was displayed at 60 Hz in a NVIS (http://www.nvisinc.com/product.html) SX111 head-mounted-display (HMD) which has a 1280 × 1024 pixels resolution and a field of view of 76° × 64° per eye, with a total viewing real estate covering 102° horizontal by 64° vertical, totalling 111 degrees across the diagonal with 50° overlap in the horizontal axis. Visual calibration of the HMD screens was carried out using the method proposed by Grechkin and colleagues^64^. Head tracking was performed by an Intersense IS-9006 (http://www.intersense.com/pages/20/14) hybrid inertial/acoustic six-degrees-of-freedom tracking system with the device attached on the top of the HMD. Head rotation data were streamed over a Virtual-Reality Peripheral Network (VRPN) network^65^ to the PC running the application.

**Figure 3:**
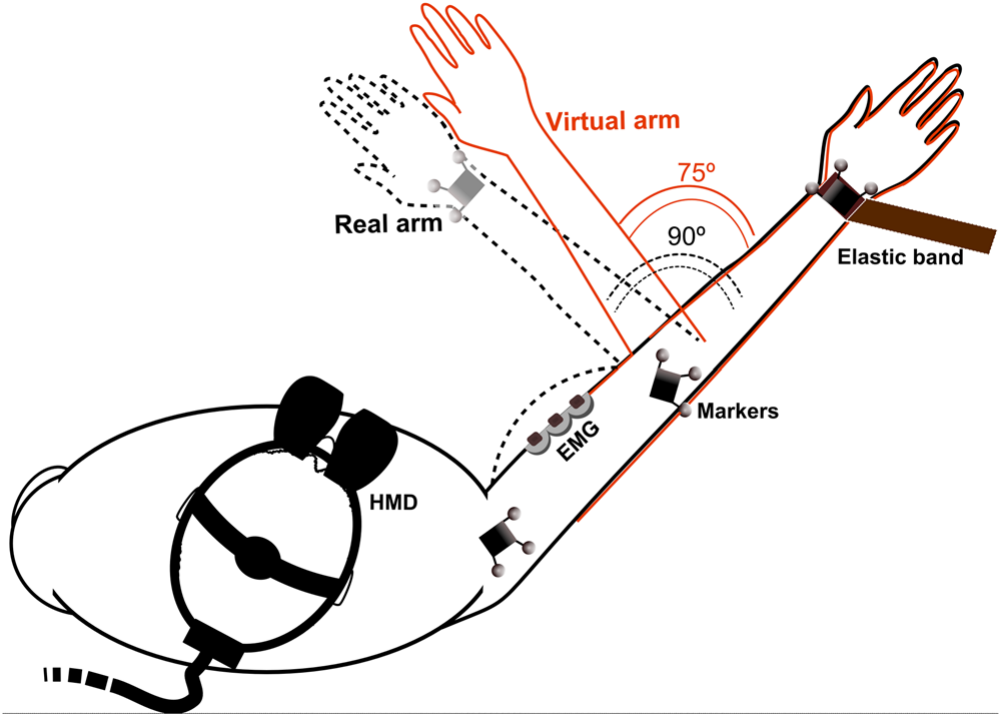
Experimental setup: three sets of reflective markers were placed along the participants arm (on the wrist, elbow and shoulder) allowing the tracking system to translate the real arm movements into those of the avatar. EMG sensors were placed on the biceps muscle to record the muscular activity while participants were pulling the elastic band. Participants were wearing an HMD whereby they saw their virtual body (they could not see their real body). Participants were instructed to always perform a movement of 90°. At the starting position, both the real (unseen) arm and the virtual arm were perfectly collocated (no misalignment). However, when the real (unseen) arm was at 90° from the original position, the virtual one was either at 75°, thus displaced −15° with respect to the real arm (“C −15°” condition); not displaced (“C 0°” condition); or displaced + 15° (“C 15°” condition).

Participants’ arm movements were tracked by a Natural Point’s Optitrack (http://www.naturalpoint.com/optitrack/) infrared system, composed of 12 cameras, tracking 3 triplets of reflective markers each one tied respectively to the shoulder, elbow and wrist of the participants (Fig. 3). Position and orientation of the markers were calculated in real time and streamed to the virtual reality application using Natural Point’s Tracking Tools software. This setup allowed the arm of the virtual character representing a participant in the VE to move accordingly with its real movements as described in a previous study^66^.

To monitor muscular activity, the EMG signal was recorded from the right biceps using a bipolar single strip electrode montage (the reference electrode was settled between the active ones). The signal was amplified [g.tec (http://www.gtec.at/content/view/full/2) gUSBAmp], digitized at 256 Hz, and filtered with a Root-Mean-Square filter (window: 10 samples). Recording and filtering was done using Matlab (http://www.mathworks.com) Simulink.

### Procedure

Participants were asked to sit down on a chair placed in the motion tracking area of the lab. Their right elbow rested on a support, to avoid muscular fatigue and improve recording consistency during the arm movements. To measure the arm movements, participants were equipped with three Optitrack trackers placed along the participant’s right arm (shoulder, elbow and wrist). An elastic band was tied to their right wrist, exerting a resistance force starting after the beginning of the movement (the elastic band was taut at 0° but exerting no force on the resting arm). The role of the elastic band was to increasingly engage the biceps muscle as flexion of the elbow increased. Three EMG electrodes were attached to their right biceps to gauge muscular activity throughout the participants’ arm movements (see Fig. 3). The experiment comprised a brief preparation phase and a subsequent experimental phase.

### Preparation/baseline phase

Participants were asked to make their arm coincide with the starting position of the virtual avatar’s arm; that is, with their elbow fully extended and their shoulder horizontally abducted to 45° (Fig. 3). Once ready, and before donning the HMD, the EMG baseline was recorded: participants were asked to repeatedly flex their elbow from the initial position (elbow straight) to an end position where the forearm reached of 90° with respect to their upper arm, and back again to starting position (Fig. 3). During the movements they were asked to focus their attention on the moving hand. The movements were repeated 15 times. The rhythm of the movements was driven by a digital metronome beeping at 0.67 Hz and kept constant for all trials and conditions. To isolate participants from noise disturbances, pink noise was played through loudspeakers at 65 dB throughout the experiment. The metronome’s beep was kept clearly audible in the pink noise.

### Experimental phase

After the preparation phase, participants donned the HMD to be visually immersed in an empty virtual room and then embodied in a gender-matched virtual body seen from a first-person perspective. The avatar was seated like the participant and reproduced his/her right arm movements in real-time, as transmitted by the tracking system, using an inverse kinematics algorithm. It has been shown that such synchronous visuomotor correlation between the real and the virtual arm movements is sufficient to induce the illusion of both ownership and agency over the virtual arm^41^. To ensure that participants were attentive and looking at the avatar’s arm throughout the task, a small virtual disk was rendered on top of the avatar’s right wrist and changed its colour randomly between 3.5 and 7 seconds. Participants were asked to verbally report the colour as soon as it changed. Before the start of the experiment all participants were tested to make sure that they could identify each colour correctly. All participants underwent three different experimental VR conditions (within-participants design): “C 0°”, “C 15°”, “C−15°”, plus one “no VR” condition that served as a baseline (described above). The three VR conditions only differed in the drift of the virtual arm compared to the real one, when the latter was flexed to 90°. The drift angle was not constant but increasing along the movement of the forearm: it was null when the elbow was straight and was either of 0°, 15° or −15° when the real elbow was at the final position (flexed to “90°”). Therefore, in “C 15°” the virtual arm was 15° ahead of the real arm, such that when the real elbow was flexed to 75°, the virtual arm appeared flexed to 90°. And in “C −15°” when the real arm was flexed to 105°, the virtual arm appeared flexed to 90°. Thus, when the real arm was at 90°, the virtual arm was either at 90°, 105° or 75° respectively. In all conditions, participants were asked to maintain their attention on the avatar’s arm throughout the condition while flexing their elbow to 90°. Particular emphasis was given to their performance and they were asked to be as precise as possible. These instructions were given as a reminder at the beginning of each condition. To keep the visual manipulation covert, they were not informed that there were three different conditions but, instead, they were told that the experiment included two brief interruptions, which were introduced to allow them to rest and to record the subjective ratings. Each condition was constituted by 15 arm movements and took 45 seconds to complete. The order of the conditions was randomized across participants.

For each condition measures of the angles reached by both the virtual and the real arm were taken, together with biceps muscular activity (EMG).

At the end of each condition participants had to verbally rate the subjective level of ownership and agency they felt over the virtual arm. Each question (enunciated in Spanish during the experiment) was scored according to a 7-point Likert Scale, 1 representing “Not at all” and 7 “Totally”. The questions were as follows:

Ownership: “Did you feel as if the right virtual arm was your own right arm?”

Agency: “Did you feel as if the arm movements were caused by your own movements?”

The order of the questions was randomized among conditions and participants.

### Data analysis

For each condition the amplitude of the real arm movement (maximum angles reached for each movement, in degrees) and the EMG activity (in mV) were recorded. The root mean square (RMS) of the EMG activity was calculated. Both the peaks’ amplitudes (RMS’s maximum peaks) and the area under the RMS curves, for each movement and condition, were considered. The area under the curve was obtained using a numerical integration function and was used to compare the muscular activity between the different conditions. With each movement, the angle at the maximum flexion of the elbow was measured and subsequently used to calculate the mean maximum angle. Similarly, the mean of the maximum peaks of the RMS responses recorded was also calculated. To correct for individual differences, data from the angles and the RMS were normalized with respect to the baseline condition according to the ratio: VR condition/baseline. One-way repeated-measures ANOVAs were carried out on RMS areas, RMS peaks and movement angles separately. A Greenhouse-Geisser (G-G) correction was applied whenever there was a violation of the sphericity assumption, as shown by the Mauchly test. Post-hoc analyses following one-way ANOVAs were conducted with the Bonferroni test.

Possible differences in ownership and agency ratings among conditions were checked with non-parametric Friedman ANOVAs.

## Data Availability

Data is available from the corresponding author on reasonable request.
